# Long-term administration and efficacy of oxaliplatin with no neurotoxicity in a patient with rectal cancer: Association between neurotoxicity and the GSTP1 polymorphism

**DOI:** 10.3892/ol.2014.1890

**Published:** 2014-02-18

**Authors:** HIRONORI KITADE, TAKEO SHIMASAKI, SAYA IGARASHI, HIROSHI SAKUMA, MITSUE MORI, NAOHISA TOMOSUGI, MASUO NAKAI

**Affiliations:** 1 Department of Pharmacy, Houju Memorial Hospital, Nomi, Ishikawa 923-1226, Japan; 2 Outpatient Cancer Chemotherapy Center, Houju Memorial Hospital, Nomi, Ishikawa 923-1226, Japan; 3 Medicalm Research Institute, Kanazawa Medical University, Kahoku, Ishikawa 920-0293, Japan; 4 Department of Radiology, Houju Memorial Hospital, Nomi, Ishikawa 923-1226, Japan; 5 Department of Surgery, Houju Memorial Hospital, Nomi, Ishikawa 923-1226, Japan

**Keywords:** oxaliplatin, neurotoxicity, colorectal cancer, GSTP1

## Abstract

Neurotoxicity is one of the most frequent side-effects of oxaliplatin. Oxaliplatin-induced cumulative and dose-limiting neurotoxicity either results in dose reduction or decreases the patients’ quality of life. However, the symptoms of neurotoxicity often vary among patients. The current study presents the case of a male with rectal cancer, who was administered a cumulative oxaliplatin dose of >5,000 mg/m^2^ without developing neurotoxicity or allergic reactions. Consequently, this patient continued therapy with modified 5-fluorouracil, leucovorin and oxaliplatin treatment for four years, with stabilization of the disease. This case indicates that if oxaliplatin-containing chemotherapy shows efficacy with no toxicity, the long-term administration of oxaliplatin would be effective and tolerable. Previously, the analysis of genomic polymorphisms in drug target genes has been important for explaining interindividual variations in the efficacy and toxicity of anti-cancer drugs. In the present patient, the glutathione S-transferase P1 (GSTP1) gene polymorphism, which is involved in the detoxification of platinum drugs, was analyzed. The genotype of the present case has been revealed as wild type (Ile/Ile) genotype. In addition, the associations between oxaliplatin-induced neurotoxicity and the GSTP1 polymorphism were also assessed. Certain studies have demonstrated that oxaliplatin-induced neurotoxicity occurs more frequently in patients with the Ile/Ile genotype, while others have demonstrated that those patients with the Val/Val or Ile/Val genotypes are more likely to develop neurotoxicity. Therefore, correlation between the GSTP1 polymorphism and oxaliplatin-induced neurotoxicity remains controversial. Overall, further development of individualized chemotherapy with an analysis of genomic polymorphisms in the drug target genes is required for the prophylaxis oxaliplatin-induced neurotoxicity.

## Introduction

Oxaliplatin is an effective agent for the adjuvant or palliative treatment of patients with colorectal cancer (CRC) (1–3). Oxaliplatin frequently causes neurotoxicity, which often results in dose reduction or treatment discontinuation. In total, 12 to 18% of patients suffer from chronic and cumulative neurotoxicity when the cumulative oxaliplatin dose reaches 800 mg/m^2^ (1), whereas other patients receive high doses of oxaliplatin without developing neurotoxicity. Therefore, elucidation of the predictive factors and pathogenetic mechanism of oxaliplatin-induced neurotoxicity is required to optimize the oxaliplatin dose for CRC patients. The current study reports the case of a patient with metastatic rectal cancer, who was administered a cumulative oxaliplatin dose of >5,000 mg/m^2^ during treatment with a modified 5-fluorouracil (5-FU), leucovorin and oxaliplatin (mFOLFOX6) regimen without experiencing neurotoxicity. In addition, the result of an analysis of the glutathione S-transferase P1 (GSTP1) gene polymorphism, which has been shown to be associated with oxaliplatin-induced neurotoxicity, is also reported. Previously published studies on the association between the GSTP1 polymorphism and oxaliplatin-induced neurotoxicity are also reviewed.

## Case report

### Patient

**Figure 1:**
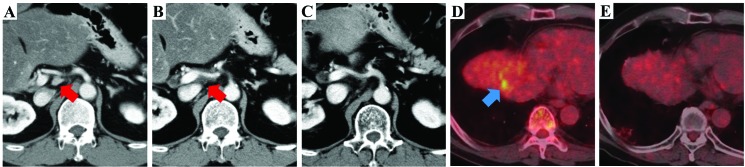
(A) Local lymph node metastases (red arrow) were detected along the CHA on CT scan. (B) Subsequent to switching from mFOLFOX6 treatment to TS-1, the recurrent tumor (red arrow) was identified again around the CHA. (C) Although the recurrent tumor disappeared subsequent to restarting treatment with mFOLFOX6, (D) the recurrent tumor (blue arrow) was revealed in the right subphrenic area on PET/CT. (E) The right subphrenic tumor disappeared following local radiation therapy and treatment with panitumumab and mFOLFOX6. CHA, common hepatic artery; CT, computed tomography; PET, positron emission tomography; mFOLFOX6, modified modified 5-fluorouracil, leucovorin and oxaliplatin regimen.

**Figure 2:**
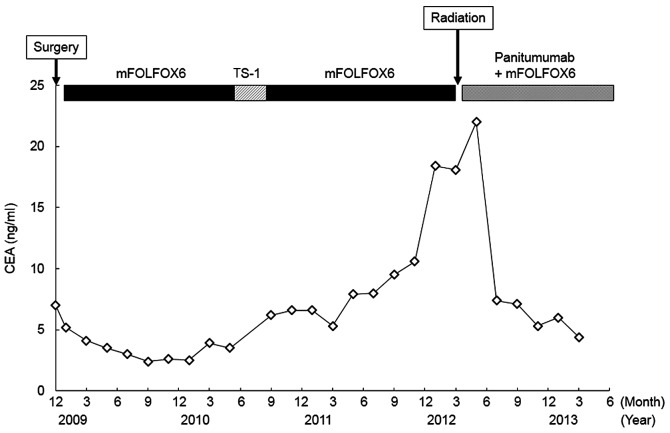
Treatment course and change in serum carcinoembryonic antigen (CEA) level. mFOLFOX6, modified 5-fluorouracil, leucovorin and oxaliplatin regimen.

A 57-year-old male who was diagnosed with rectal cancer accompanied by bladder invasion, underwent resection of the locally invasive, primary tumor (T4N0M0, stage II). Approximately three years after surgery, the carcinoembryonic antigen (CEA) level of the patient increased and a computed tomography (CT) scan revealed a recurrent tumor in the right lobe of the liver. The patient then underwent surgery to remove the recurrent liver metastasis and was treated intravenously (IV) with FOLFOX4 (200 mg/m^2^ leucovorin, 400 mg/m^2^ 5-FU bolus and 600 mg/m^2^ 5-FU over 22 h on days 1 and 2, and an additional 85 mg/m^2^ oxaliplatin on day 1, every two weeks) as adjuvant chemotherapy for four cycles. Approximately two years later, the CEA level of the patient increased again and lymph node metastases were identified along the common hepatic artery (CHA) on the CT scan (Fig. 1A). Local excision was performed for the recurrent lesion, and the patient began IV treatment with mFOLFOX6 (85 mg/m^2^ oxaliplatin, 200 mg/m^2^ leucovorin, 400 mg/m^2^ 5-FU bolus on day 1 and 2,400 mg/m^2^ 5-FU over 46 h every two weeks) as adjuvant chemotherapy. As the patient requested long-term adjuvant therapy, mFOLFOX6 was administered for 18 months without recurrence. Subsequent to this, TS-1 (Taiho Pharmaceutical, Tokyo, Japan) was initiated at a dosage of 60 mg twice daily on days 1 to 28 every six weeks following a discussion with the patient. However, the CEA level of the patient increased and the recurrent tumor around the CHA showed progression on the CT scan (Fig. 1B). Next, treatment with mFOLFOX6 was restarted and continued for 17 months without progressive disease (Fig. 1C). However, the CEA level once again increased and the recurrent tumor was detected in the right subphrenic area on positron emission tomography/CT; this was treated by local radiation therapy (Fig. 1D). Panitumumab (6 mg/kg every two weeks) was added to the mFOLFOX6 regimen, and the patient continued the therapy (Fig. 1E). Although the patient was administered ~5,000 mg/m^2^ oxaliplatin during the clinical course, the treatment was safe and tolerated without causing neurotoxicity (Fig. 2).

Written informed consent was obtained from the patient, and the analysis was approved by the Institutional Ethics Committee of Houju Memorial Hospital (Nomi, Japan).

### Genomic analysis

All genomic analyses were carried out by Falco Biosystems Ltd. (Kyoto, Japan). Genomic DNA was extracted from whole blood using a QIAamp DNA Blood Mini kit (Qiagen, Valencia, CA, USA). The DNA sample was analyzed using polymerase chain reaction (PCR). The PCR reaction contained 50 ng genomic DNA, 25 μl 2X PCR Master Mix (Promega, Madison, WI, USA) and 100 μM of each primer. The PCR conditions were 94°C for 2 min, followed by 35 cycles of 94°C for 1 min, 55°C for 30 sec, 72°C for 1 min and then 72°C for 10 min. The PCR products were purified using the Agencourt AMPure XP kit (Beckman Coulter, Inc., Miami, FL, USA), and the sequence reactions of the purified products were then performed using the BigDye Terminators v1.1 Cycle Sequencing kit (Applied Biosystems, Foster City, CA, USA). Cycle conditions were 96°C for 1 min, followed by 25 cycles of 96°C for 10 sec, 50°C for 5 sec and 60°C for 4 min. The reaction products were purified using the Wizard Magnesil Sequencing Reaction Clean-Up System (Promega) for the elimination of Dye Terminator. The purified products underwent capillary electrophoresis using Genetic Analyzer 3130xl (Applied Biosystems). Data were analyzed using Sequencing Analysis Software (Applied Biosystems).

### Results

**Figure 3:**
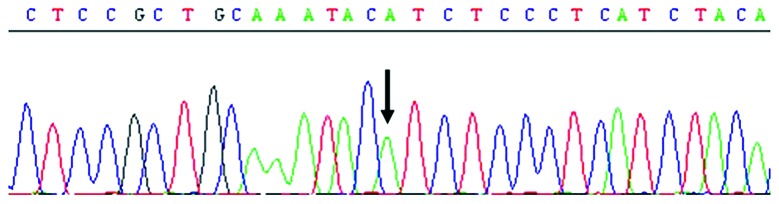
Glutathione S-transferase P1 (GSTP1) polymorphism of present case.

A single nucleotide polymorphism at position 313 of exon 5 in the GSTP1 gene leads to isoleucine/valine substitution (4). GSTP1 genotype analysis demonstrated that the patient was found to possess the wild type (Ile/Ile) genotype, as the GSTP1 polymorphism was homozygous for A/A at position 313 of exon 5 in amino acid codon 105 (Fig. 3).

## Discussion

Oxaliplatin-based chemotherapy has become a standard regimen for CRC, as an adjuvant treatment and as a treatment for advanced disease (1–3). Neurotoxicity is the major dose-limiting toxicity of oxaliplatin, and it greatly affects the continuation of therapy and the patients’ quality of life. Although there have been several studies on the prevention of oxaliplatin-related neurotoxicity, including the use of ‘Stop and Go’ therapy, Ca/Mg infusions and goshajinkigan, no recommendation has been made for the prophylaxis of oxaliplatin-induced neurotoxicity (5–7). However, it is noteworthy that there was no neurotoxicity despite the efficacious long-term, high-dose administration of oxaliplatin in the present case.

It has been indicated that oxaliplatin-induced neurotoxicity is caused by alteration of the voltage-gated sodium channels by its metabolite, oxalate (8,9). In addition, pharmacogenetics is becoming extremely important in the prediction of toxicity and response (10). The GSTs are a multigene family of enzymes that catalyze the conjugation of glutathione to electrophilic xenobiotics and facilitate their excretion from the body (4). The enzymes have at least five major classes. Among them, GSTP1, which is highly expressed in CRC, participates in the detoxification of platinum drugs and may be involved in the resistance to platinum-based chemotherapy (11,12). A single nucleotide polymorphism (A>G) at position 313 of exon 5 causes an isoleucine to valine substitution in amino acid codon 105 (Ile105Val) in the GSTP1 gene (4). Several studies have shown an association between the GSTP1 polymorphism and oxaliplatin efficacy and toxicity (Table I). Certain studies have reported longer progression-free survival (PFS) or overall survival (OS) times in patients with a homozygous (Val/Val) or heterozygous (Ile/Val) genotype when treated with oxaliplatin (13–15). While it has been found that cumulative neurotoxicity is more common in patients with the Ile/Ile genotype (16–18), certain studies have shown that neurotoxicity is more frequent in patients with the Val/Val or Ile/Val genotypes (14,15,19–21). However, other studies found no correlation between PFS, OS or neurotoxicity and the GSTP1 genotype (18,20,22–24). As shown in Fig. 3, the present patient possessed the Ile/Ile genotype. It is thought that the GSTP1-105Ile protein could enhance oxaliplatin-induced neurotoxicity through inhibition of c-Jun NH2-terminal kinase (JNK), whereas the Val variant shows higher JNK activity, thereby increasing the expression of genes involved in cellular defense, and thus possibly protecting the cells against platinum-induced toxicity (25). The association between the GSTP1 polymorphism and oxaliplatin efficacy and toxicity remains controversial.

In conclusion, the current study describes the case of a patient with rectal cancer, who safely received long-term treatment with oxaliplatin. It has been reported that administration of the three active agents, 5-FU/leucovorin, irinotecan and oxaliplatin, is associated with prolonged OS in advanced CRC (26). When specific anti-tumor agents show efficacy in patients, it is important that adverse events are prevented properly in order to continue the therapy for long periods. Further development of individualized chemotherapy with an analysis of genomic polymorphisms in the drug target genes, including GSTP1, is required to prevent oxaliplatin-induced cumulative neurotoxicity.

## Figures and Tables

**Table I:** Overview of published studies on GSTP1 polymorphism and efficacy or neurotoxicity of oxaliplatin among patients with gastrointestinal cancers.

First author (ref.)	Year	Sample size, n	Cancer type	Regimen	Genotype associated with longer PFS time	Genotype associated with longer OS time	Genotype associated with frequent neurotoxicity
Stoehlmacher J *et al* (13)	2002	107	CRC	5-FU/L-OHP	-	A/G or G/G	-
Grothey A *et al* (19)	2005	299	CRC	FOLFOX4	-	-	A/G or G/G
Lecomte T *et al* (16)	2006	64	CRC, GC, PC	FOLFOX4 (72%)	-	-	A/A
Gamelin L *et al* (22)	2007	122	CRC	FOLFOX	-	-	Not significant
Ruzzo A *et al* (20)	2007	166	CRC	FOLFOX4	Not significant	-	A/G or G/G
Paré L *et al* (17)	2008	126	CRC	5-FU/L-OHP	-	-	A/A
Kweekel DM *et al* (23)	2009	56	CRC	XELOX	Not significant	Not significant	Not significant
Goekkurt E *et al* (18)	2009	134	GC	FLO or FLP	Not significant	Not significant	A/A
Chen YC *et al* (14)	2010	166	CRC	FOLFOX4	A/G or G/G	A/G or G/G	A/G or G/G
Kanai M *et al* (21)	2010	82	CRC	mFOLFOX6	-	-	A/G or G/G
Inada M *et al* (24)	2010	51	CRC	mFOLFOX6	-	-	Not significant
Hong J *et al* (15)	2011	52	CRC	SOX	A/G or G/G	-	A/G or G/G

PFS, progression free survival; OS, overall survival; CRC, colorectal cancer; GC, gastric cancer; PC, pancreatic cancer; 5-FU, 5-fluorouracil; L-OHP, oxaliplatin; FOLFOX4, biweekly treatment with 200 mg/m^2^ leucovorin, 400 mg/m^2^ 5-FU bolus and 600 mg/m^2^ 5-FU over 22 h on days 1 and 2, and 85 mg/m^2^ oxaliplatin on day 1; XELOX, triweekly treatment with 130 mg/m^2^ oxaliplatin and 1,000 mg/m^2^ capecitabine twice daily on days 1 to 14; mFOLFOX6, biweekly treatment with 85 mg/m^2^ oxaliplatin, 200 mg/m^2^ leucovorin, 400 mg/m^2^ 5-FU bolus on day 1 and 2,400 mg/m^2^ 5-FU over 46 h; FLO, biweekly treatment with 85 mg/m^2^ oxaliplatin, 200 mg/m^2^ leucovorin and 2,600 mg/m^2^ 5-FU; FLP, biweekly treatment with 50 mg/m^2^ cisplatin, weekly treatment with 200 mg/m^2^ leucovorin and 2,000 mg/m^2^ 5-FU; SOX, biweekly treatment with 85 mg/m^2^ oxaliplatin and 40 mg/m^2^ TS-1 twice daily on days 1 to 7.
